# Supplementary material for: Myofiber stretch induces tensile and shear deformation of muscle stem cells in their native niche
Source: Biophys J. 2021 Jun 2;120(13):2665–78. doi: 10.1016/j.bpj.2021.05.021 (PMC8390894; doi:10.1016/j.bpj.2021.05.021)
Supplement: Document S1. Fig. S1 [file mmc1.pdf]

Biophysical Journal, Volume *120*

Supplemental information

Myofiber stretch induces tensile and shear deformation of muscle stem cells in their native niche

Mohammad Haroon, Jenneke Klein-Nulend, Astrid D. Bakker, Jianfeng Jin, Hadi Seddiqi, Carla Offringa, Gerard M.J. de Wit, Fabien Le Grand, Lorenzo Giordani, Karen J. Liu, Robert D. Knight, and Richard T. Jaspers

Supplemental Information

MS title: Myofiber stretch induces tensile and shear deformation of muscle stem cells in their native niche

Authors: M Haroon, J Klein-Nulend, AD Bakker, J Jin, H Seddiqi, C Offringa, GMJ de Wit, F Le Grand, L Giordani, KJ Liu, RD Knight, RT Jaspers

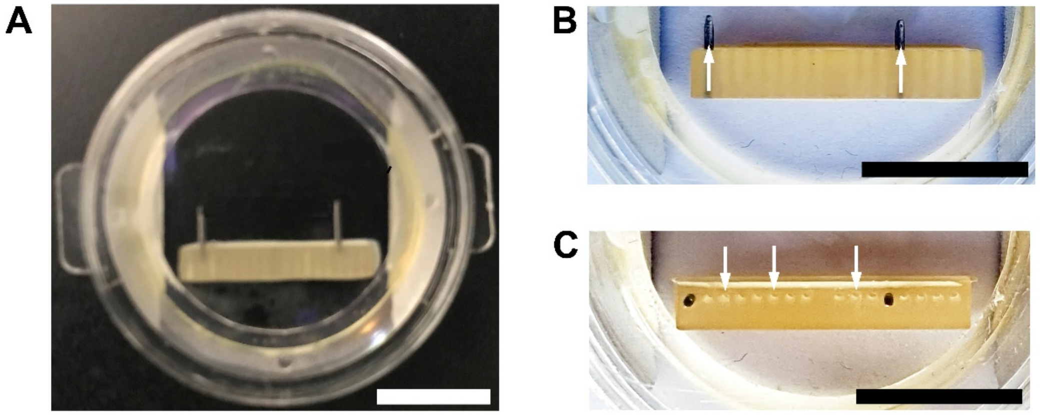

Figure S1. Chamber for incubation of myofiber bundle at slack length or high strain, consisting of a glass bottom and adjustable platinum rods. (A) Chamber used to maintain the myofiber bundle at slack length or high strain. (B) The platinum rods (arrows) in the chamber used to mount the myofiber bundles. (C) The inserts (arrows) in the bar holder allow to maintain the myofiber bundle at slack length or high strain by adjusting the distance between the rods. Scale bar, 1 cm.
