# Supplementary material for: Myofiber stretch induces tensile and shear deformation of muscle stem cells in their native niche
Source: Biophys J. 2021 Jun 2;120(13):2665–78. doi: 10.1016/j.bpj.2021.05.021 (PMC8390894; doi:10.1016/j.bpj.2021.05.021)
Supplement: Document S2. Article plus supporting material [file mmc2.pdf]

Biophysical Journal
Article

Myofiber stretch induces tensile and shear deformation of muscle stem cells in their native niche

Mohammad Haroon,[1] Jenneke Klein-Nulend,[2] Astrid D. Bakker,[2] Jianfeng Jin,[2] Hadi Seddiqi,[2] Carla Offringa,[1] Gerard M. J. de Wit,[1] Fabien Le Grand,[3] Lorenzo Giordani,[4] Karen J. Liu,[5] Robert D. Knight,[5,*] and Richard T. Jaspers[1,*]

[1]Laboratory for Myology, Faculty of Behavioural and Movement Sciences, Vrije Universiteit Amsterdam, Amsterdam Movement Sciences, Amsterdam, the Netherlands; [2]Department of Oral Cell Biology, Academic Centre for Dentistry Amsterdam, University of Amsterdam and Vrije Universiteit Amsterdam, Amsterdam Movement Sciences, Amsterdam, the Netherlands; [3]Faculty of Medicine and Pharmacy, NeuroMyoGène UCBL, CNRS UMR 5310, INSERM U1217, Lyon, France; [4]Sorbonne Université, INSERM UMRS974, Center for Research in Myology, Paris, France; and [5]Centre for Craniofacial and Stem Cell Biology, Guy's Hospital, King's College London, London, United Kingdom

ABSTRACT Muscle stem cells (MuSCs) are requisite for skeletal muscle regeneration and homeostasis. Proper functioning of MuSCs, including activation, proliferation, and fate decision, is determined by an orchestrated series of events and communication between MuSCs and their niche. A multitude of biochemical stimuli are known to regulate MuSC fate and function. However, in addition to biochemical factors, it is conceivable that MuSCs are subjected to mechanical forces during muscle stretch-shortening cycles because of myofascial connections between MuSCs and myofibers. MuSCs respond to mechanical forces in vitro, but it remains to be proven whether physical forces are also exerted on MuSCs in their native niche and whether they contribute to the functioning and fate of MuSCs. MuSC deformation in their native niche resulting from mechanical loading of ex vivo myofiber bundles was visualized utilizing *mT/mG* double-fluorescent Cre-reporter mouse and multiphoton microscopy. MuSCs were subjected to 1 h pulsating fluid shear stress (PFSS) with a peak shear stress rate of 6.5 Pa/s. After PFSS treatment, nitric oxide, messenger RNA (mRNA) expression levels of genes involved in regulation of MuSC proliferation and differentiation, ERK 1/2, p38, and AKT activation were determined. Ex vivo stretching of extensor digitorum longus and soleus myofiber bundles caused compression as well as tensile and shear deformation of MuSCs in their niche. MuSCs responded to PFSS in vitro with increased nitric oxide production and an upward trend in *iNOS* mRNA levels. PFSS enhanced gene expression of *c-Fos*, *Cdk4*, and *IL-6*, whereas expression of *Wnt1*, *MyoD*, *Myog*, *Wnt5a*, *COX2*, *Rspo1*, *Vangl2*, *Wnt10b*, and *MGF* remained unchanged. ERK 1/2 and p38 MAPK signaling were also upregulated after PFSS treatment. We conclude that MuSCs in their native niche are subjected to force-induced deformations due to myofiber stretch-shortening. Moreover, MuSCs are mechanoresponsive, as evidenced by PFSS-mediated expression of factors by MuSCs known to promote proliferation.

SIGNIFICANCE Skeletal muscle homeostasis and regeneration involves the proper function of adult muscle stem cells (MuSCs). It is well known that muscle regeneration requires an orchestrated biochemical communication between MuSCs and host myofibers. However, it is unknown whether biophysical cues are equally important and whether MuSCs in vivo are subjected to mechanical loads. This study provides new, to our knowledge, important insight into the effect of biophysical forces on MuSCs in situ and shows that mechanical forces can influence MuSC function. This stimulates further research on the role of mechanical cues in determining MuSC function, muscle regeneration, and age-related or disease-related alterations in these biophysical cues and mechanotransduction.

INTRODUCTION

Skeletal muscle regeneration requires activation and proliferation of muscle stem cells (MuSCs). During organismal aging, the number of MuSCs is reduced, and MuSCs lose their proliferation potential (1). Moreover, muscle pathologies characterized by impaired regeneration by MuSCs

Submitted August 12, 2020, and accepted for publication May 18, 2021.

*Correspondence: robert.knight@kcl.ac.uk or r.t.jaspers@vu.nl

Editor: Cynthia Reinhart-King.

https://doi.org/10.1016/j.bpj.2021.05.021

© 2021 Biophysical Society.

This is an open access article under the CC BY license (http://creativecommons.org/licenses/by/4.0/).

lead to atrophy and fibrosis (2). In young, uninjured muscles, myofiber hypertrophy is accompanied by MuSC activation, proliferation, and subsequent fusion with the host myofiber to increase myonuclei numbers (3). This ensures that the myonuclear domain does not exceed a critical size (3). Understanding the mechanisms underlying MuSC function is required for adequate strategies to maintain MuSC self-renewal and regenerative function.

During regeneration, a multitude of growth factors and cytokines are secreted by myofibers, neighboring immune cells, and fibroblasts, which activate MuSCs and stimulate proliferation, differentiation, and self-renewal (4). During strenuous exercise, myofibers are subjected to large stretch-shortening deformations by active contraction of myofibers and passive external loads applied to myofibers from antagonist muscle activity. Such mechanical loading stimulates the expression of growth factors and cytokines within the myofiber microenvironment (5). These growth factors and cytokines act in a paracrine or endocrine manner on the MuSCs and thus regulate their function (6,7). This indicates that mechanical loads are essential for MuSC regenerative function, but little is known about how MuSCs themselves respond to mechanical force in vivo.

Skeletal muscle fibers have the ability to sense mechanical cues via transmembrane complexes and stretch-activated calcium channels (5). Three-dimensional (3D) finite element modeling (FEM) of muscle revealed that global strain application onto a muscle in vivo causes high local tensile strains within the extracellular matrix (ECM) (8). The contractile apparatus within the myofiber is also exerting force laterally (9), and FEM suggests that these lateral forces cause shear deformations of the ECM by slippage along a plane parallel to the imposed stress (10). These deformations cause interstitial fluid movement within the muscle (11), and currently it is unknown whether MuSCs can sense these fluid movements. Moreover, force-induced ECM deformations could also be transmitted to the MuSCs, which are anchored to the sarcolemma of the myofiber by cadherins, and on their apical side to the basal lamina of the endomysium via glycocalyx, integrins, syndecans, dystroglycans, and sarcoglycans (12). Therefore, it has been suggested that MuSCs experience tensile strain and shear stress after muscle tissue contraction (13). Whether mechanical forces do induce strain and stress onto MuSCs in muscle has yet to be explored. In vitro studies have shown that MuSCs are sensitive to tensile forces (14,15), however, it is unknown whether MuSCs within their native niche are subjected to mechanical forces resulting in cell deformation. In this study, we tested whether physical load induces deformation of MuSCs in their native niche using isolated mouse myofiber bundles containing an intact endomysium and basal lamina. We were able to show that upon myofiber bundle stretch, the MuSCs undergo substantial shear and tensile strain deformation.

Because MuSCs in their niche are mechanically loaded, which causes interstitial fluid movement within the muscle (11), the question arises whether they are able to respond to fluid-shear-induced mechanical loads by changing the expression of factors involved in MuSC self-renewal, proliferation, and differentiation. Bone cells are subjected to shear forces because of the interstitial fluid flow within the canaliculi of bones during mechanical loading, which are estimated to be 0.8–3 Pa (16). Bone cells respond to these shear forces by prostaglandin production and nitric oxide (NO) upregulation. Moreover, in bone cells, shear forces activate members of the mitogen-activated protein kinases (MAPKs), including the extracellular signal-regulated kinase 1/2 (ERK1/2) and p38 MAPK (17,18). These kinases are central players in signal transduction pathways and involved in the regulation of transcription factor activity, thereby affecting cellular behavior including proliferation, differentiation, and cell survival (19). ERK1/2 affects cell proliferation by regulating, among others, cyclin D1 transcription via Fos and Myc proteins (20,21). p38 MAPK, however, is a well-known regulator of proinflammatory cytokine synthesis (22). p38 MAPK is activated in response to cellular stress, i.e., osmotic pressure, oxidative stress, and cytokines (23,24). In addition, fluid flow induces osteoblast proliferation via phosphatidylinositol 3-kinase and the AKT pathway (25). Activation of phosphatidylinositol 3-kinase results in recruitment of AKT to the plasma membrane and its phosphorylation and activation (26). Activated AKT acts on various downstream targets and affects cellular function, i.e., proliferation, metabolism, and cell survival (27). However, the effect of shear forces on MAPK and AKT signaling in MuSCs is still unexplored. Mechanical loading of myofibers has been shown to increase NO production and regulate NO synthase (NOS) expression and activity (28). Pulsating fluid shear stress (PFSS) increases NO production and expression of interleukin-6 (*IL-6*) and cyclooxygenase-2 (*COX2*) in myotubes (29). NO plays a role in Vang-like protein 2 (Vangl2) expression in MuSCs, resulting in enhanced MuSC self-renewal during skeletal muscle regeneration (30). Noncanonical Wnt signaling plays a crucial role in muscle regeneration (31), and mechanical loading is known to activate Wnt signaling in bones (32). Mechanical cues, such as tensile strain and shear stress, constitute a physiologically relevant and novel, to our knowledge, mechanism that can therefore potentially activate Wnt/planar cell polarity (PCP) signaling, thereby promoting MuSC self-renewal. We therefore investigated whether mechanical loads on MuSCs induce NO production and expression of biochemical factors that are crucial for MuSC regenerative function. We show here that mechanical loading induced NO production in MuSCs and upregulated *COX2*, *IL6*, and *Cdk4* messenger RNA (mRNA) expression levels. Moreover, FEM showed that PFSS treatment induced fluid pressure, fluid shear stress, and tensile stress on MuSCs. PFSS

also induced activation of members of the MAPK signaling pathway, i.e., ERK 1/2 and p38.

MATERIALS AND METHODS

Transgenic mice and myofiber bundle isolation

Animal procedures were conducted according to the UK Home Office project license P8D5E2773 (K.J.L.) and the guidelines of the European Community and French Veterinary Department, approved by the Sorbonne Université Ethical Committee for Animal Experimentation. Paired box 7 (Pax7) creERT2 mice (Jackson strain: Pax7$^{tm2.1(cre/ERT2)Fan}$) on a mixed C57BL6/CD1 background were crossed with *mTmG* reporter mice (Jackson strain: Gt(ROSA)26Sor$^{tm4(ACTB\text{-}tdTomato,\text{-}EGFP)Luo}$). Targeted deletion in the CreERT animals was performed by intraperitoneal tamoxifen injection (150 mg/kg body weight) at 40 and 20 h before euthanasia. 20 mg/mL tamoxifen solution was prepared by dissolving 10 mg tamoxifen in 100 μL 100% ethanol and adding 900 μL corn oil. Compound heterozygous transgenic mice (15–17 months, n = 3) were used for in situ mechanical loading of MuSCs and image acquisition as described later. Mice were euthanized, and extensor digitorum longus (EDL) and soleus (SO) muscles extracted. Both EDL and SO muscles were dissected for isolation of myofiber bundles, consisting of ~20 myofibers, using fine-tipped forceps and scissors under a dark-field illuminated microscope. The EDL and SO fibers were cut longitudinally. This damages the perimysium surrounding the fascicles, but the basal laminae surrounding the myofibers within the myofiber bundle stay intact. Moreover, imaging was performed on MuSCs within the myofiber bundles to avoid imaging of the outermost fibers that might contain damaged basal laminae.

Primary MuSC isolation and fluorescence-activated cell sorting

Primary MuSCs were isolated from young (2 months; n = 4) male mouse (C57BL/6J) hindlimb muscles (hamstring muscle group, quadriceps, tibialis, EDL, gastrocnemius, SO, gluteus) after enzymatic digestion, followed by fluorescence-activated cell sorting purification. Briefly, after dissection, muscles were first digested with collagenase II (1000 U/mL; Worthington Biochemical, Lakewood, NJ) in Ham's F10 containing 10% horse serum, washed, and then further digested with collagenase II (1000 U/mL) and dispase (11 U/mL) for 30 min. After digestion, cells were washed in Ham's F10 containing 10% horse serum, passed 10 times through a 20-gauge needle syringe, and then filtered with a 35-mm cell strainer (Falcon; Corning, NY). Cells were then stained with the antibodies rat CD31-eFluor450 (1/500; eBiosciences, Thermo Fisher Scientific, San Diego, CA), rat CD45-eFluor450 (1/500; eBiosciences, Thermo Fisher Scientific), rat Ly6A-FITC (SCA1) (1/500; eBiosciences, Thermo Fisher Scientific), rat CD106-PE (1/200; eBiosciences, Thermo Fisher Scientific), and rat α7 integrin-APC (1/1000; AbLab, Vancouver, Canada) and sorted using a FACS Aria II (BD Biosciences, San Jose, CA). MuSCs were isolated as CD31$^-$, CD45$^-$, Sca1$^-$, α7 integrin$^+$, and CD106$^+$.

In situ mechanical loading of MuSCs

The myofiber bundles (n = 8) were mounted at their slack length (low strain) by attaching the tendons at both ends to small rods in chambers (Bioptechs, Butler, PA; see Fig. S1) containing tyrode solution (NaCl, 7.5 g/L; KCl, 0.35 g/L; $MgCl_2 \cdot 6H_2O$, 0.214 g/L; $NaH_2PO_4 \cdot H_2O$, 0.058 g/L; $NaHCO_2$, 1.7 g/L; $CaCl_2 \cdot H_2O$, 0.2 g/L), which was saturated with a carbogen gas mixture (95% O_2 and 5% CO_2) at 37°C. To induce a high strain on the myofiber bundle, the bundle was stretched by ~40% above slack length (i.e., high strain) in accordance with the previous literature (33).

In situ image acquisition

Images were acquired on a Zeiss Axioscope (Oberkochen, Germany) using a 7MP multiphoton system with Vision II and MPX (Chameleon, Santa Clara, CA) lasers and a 20× water dipping objective (NA 1.0). The Vision II laser was tuned to 890 nm (range: 880–900 nm), and the MPX laser to 1200 nm with reflected emissions captured by non-descanned photomultiplier detectors (channel 1 and 2), GaAsP BiG detector (channel 3), or transmitted light detector (channel 4). Emission filters used were shortpass 485 nm (channels 1 and 4), bandpass 500–550 (channel 2), and bandpass 575–610 (channel 3). The signals detected were second harmonic (channel 1), GFP (channel 2), and tdTomato (channel 3). Image stacks were acquired every ~1 μm in the *Z* direction.

Image analysis

Cells were segmented in the Medical Imaging Interaction Toolkit (https://www.mitk.org/wiki/The_Medical_Imaging_Interaction_Toolkit_(MITK)) for 3D reconstruction. Analysis of cellular parameters, i.e., sarcomere length, cell length, cell width, cell aspect ratio (major axis/minor axis), and cell roundness (4 × ([area])/(π × [major axis]^2) were performed in ImageJ, version 1.52h (Wayne Rasband, National Institutes of Health, Bethesda, MD). Tensile strain was calculated by the change in length (ΔL) divided by the initial length (L_0) in the *XY* plane ($\Delta L/L_0$). Shear strain was quantified by measuring the difference in change in length (ΔL) of the two lateral sides of the cell and the change in angle between both lateral sides in the *YZ* plane (shear angle).

Cell culture

For in vitro mechanical loading by PFSS, MuSCs were expanded on matrigel (Corning, Bedford, MA)-coated culture flasks with growth medium consisting of Ham's F-10 Nutrient Mix (Gibco, Paisley, UK) supplemented with 20% fetal bovine serum (FBS; Gibco), 10 μg/mL penicillin (Sigma-Aldrich, St. Louis, MO), 10 μg/mL streptomycin (Sigma-Aldrich), and 2.5 ng/μL of recombinant human fibroblast growth factor (R&D Systems, Minneapolis, MN), and cultured at 37°C in a humidified atmosphere of 5% CO_2 in air. Upon 70% confluence, cells were harvested using 0.1% trypsin and 0.1% EDTA (Gibco) in phosphate-buffered saline.

PFSS

MuSCs were seeded at 12–20 × 10^3/cm^2 on matrigel (Corning)-coated glass slides (2.5 × 6.5 cm; n = 10) and cultured for 2–4 days. 1 h before PFSS, culture medium was refreshed by medium containing a low serum concentration (2% FBS). PFSS was applied as described earlier (29). Briefly, PFSS was generated by pumping 7 mL of culture medium through a parallel-plate flow chamber containing MuSCs. Cells were subjected to a cyclic changing pressure gradient with a peak shear stress rate of 6.5 Pa/s (pulse amplitude: 1 Pa; pulse frequency: 1 Hz). We adopted the 1 Hz frequency, which mimics the walking strain cycles (stride frequency) (34). Static control cells were kept in similar conditions as PFSS-loaded cells. After 1 h PFSS treatment or static control culture, images were taken, and RNA was isolated. To determine the number of MuSCs detached as a result of PFSS exposure, cells were counted in random images acquired pre- and post-PFSS.

Fluid shear stress and live-cell imaging of MuSC deformation

MuSCs were seeded at 15 × 10^3/cm^2 on matrigel (Corning)-coated glass slides (22 × 22 mm) and cultured for 3 days. 3 h before fluid shear stress application, the culture medium was refreshed by medium with low serum

(2% FBS) containing 250 nM live-cell stain for F-actin (SiR-Actin; Spirochrome, Stein am Rhein, Switzerland) and nucleic acid (SiR700-DNA; Spirochrome). To determine the effect of fluid shear stress on MuSC morphology, the glass slide was placed in a microfluidic chamber connected to a pump. MuSCs were then subjected to constant fluid shear stress (CFSS) of 1 Pa/s by pumping 7 mL medium and imaged using a Leica TCS SP8 confocal microscope as described (29). Cells were irradiated with a pulsed white light laser at a wavelength of 645 nm, and *XZ* time-lapse images were acquired using a 40× 1.3 NA oil objective.

FEM

A one-way fluid-structure interaction model was employed to predict and quantify pulsating fluid dynamics on an adherent MuSC and the solid mechanics of this cell in a parallel-plate flow chamber over time by using a commercial finite-element software package (Multiphysics v5.4; COMSOL, Stockholm, Sweden). Fluid was flowing over the adherent MuSC in the chamber (35). FEM was performed with a time-dependent fully coupled solver. An iterative method for the numerical solution of a nonsymmetric system of linear equations (generalized minimal residual algorithm) was used to evaluate the variable fluid pressure, fluid shear stress on the cell, and Von Mises stress and solid displacement of the cell.

Model assumptions

To perform fluid-structure interaction analysis on an adherent MuSC in a parallel-plate flow chamber, the following assumptions were made about boundary and initial conditions to simplify the mathematical modeling. The chamber was assumed as solid, i.e., incompressible and impermeable for fluid. The cell geometry was considered as half ellipsoid (volume: 2.1 mm^3; surface area: 9.4 mm^2; cell apex height: 10 μm; cell area: 314 μm^2), as the effect of cell geometry on pulsating fluid dynamics and solid mechanics was not taken into consideration. The cell was assumed to be an isotropic linear elastic material. The culture medium inside the chamber was considered as incompressible and a homogeneous Newtonian fluid. The effect of heat dissipation from the culture medium was neglected. Therefore, the culture medium specifications, such as dynamic viscosity and density, were assumed to be constant during the computational analysis. The cell was attached to the bottom surface of the chamber during the modeling period.

Initial and boundary conditions

The initial fluid velocity and average fluid pressure at the outlet surface of the parallel-plate flow chamber were set to 0. A no-slip boundary condition was applied to the inner surface of the parallel-plate flow chamber and to the outer surface of the adherent cell. As the inlet boundary condition, PFSS (inlet flow rate: 3.5 mL/min; amplitude: 1.0 Pa; frequency: 1.0 Hz) was chosen. The dynamic viscosity (μ) of the fluid was 0.78 mPa s, and the density (ρ) was 1.007 g/mL. The Young's modulus (E) of the cell was 28 kPa, the Poisson's ratio (ϑ) was 0.38, and the cell density (ρ_{cell}) was 1.06 g/mL.

Mesh generation

The model mesh was constructed with normal elements (total elements: 185,713; tetrahedra, 123,849; pyramids, 48; prisms, 61,816; triangles, 31,920; quads, 632; edge elements, 676; and vertex elements, 13) using COMSOL Multiphysics v5.4. Average element quality was 0.62 measured by skewness, which is considered as good element quality. The model mesh was achieved after a mesh convergence process based on the fluid dynamics variation (fluid pressure and fluid shear stress) as described previously (36).

Average fluid pressure, fluid shear stress, Von Mises stress, and solid displacement

The average values of fluid pressure, fluid shear stress, Von Mises stress, and solid displacement were obtained based on the following equation:

$$\theta_{ave} = \frac{\iint \theta d\Omega}{\iint d\Omega}, \tag{1}$$

where θ is the dependent variable (fluid pressure, fluid shear stress, Von Mises stress, or solid displacement), and Ω is the spatial domain, which equals the outer surface of the cell.

Nitric oxide analysis

Medium samples were taken from PFSS-treated and static cell cultures (n = 20) at intervals (10, 30, and 60 min) for NO analysis. NO production was measured as nitrite (NO_2^-) accumulation in the medium using Griess reagent, and absorbance was measured at 540 nm with a microplate reader (Bio-Rad Laboratories, Veenendaal, the Netherlands) as described earlier (29).

RNA isolation and reverse transcription

After PFSS and static treatment (n = 7–10), micrographs were taken, and cells were lysed with 700 μL Trizol (Thermo Fisher Scientific, Carlsbad, CA) and stored at −80°C overnight. Total RNA was isolated using RiboPure Kit (Applied Biosystems, Foster City, CA) and quantified (NanoDrop Technologies, Thermo Fisher Scientific, Wilmington, DE). mRNA (400 ng) was reverse transcribed to complementary DNA (cDNA) using a high-capacity RNA-to-cDNA kit (Applied Biosystems).

Quantitative real-time PCR

Real-time PCR was performed on the StepOne Real-Time PCR system (Applied Biosystems). Primers were designed using Universal Probe library from Roche Diagnostics. Data were analyzed using StepOne v2.0 software (Applied Biosystems) and normalized relative to *18S* ribosomal RNA levels. *Wnt1*, *Wnt3a*, *Wnt5a*, *Wnt7a*, *Wnt10b*, *IL-6*, *Rspo1*, *Dkk1*, *COX2*, *nNOS*, *eNOS*, and *iNOS* transcript levels were measured using Taqman qPCR using inventoried Taqman gene expression assays (Applied Biosystems). mRNA levels of *18S*, *MyoD*, *Myog* (myogenin), *c-Fos*, *Cdk4* (cyclin-dependent kinase 4), *Vangl2*, and *MGF* were measured using SYBR green (Thermo Fisher Scientific). Primer sequences used for real-time PCR were *18S* (5′ GTAACCCGTTGAACCCCATT and 5′ CCATCCAATCGGTAGTAGCG), *MyoD* (5′ CATCCAGCCCGCTCCAAC and 5′ GGGCCGCTGTAATCCATCATGCC), *Myog* (5′ CCAGCCCATGGTG CCCAGTGA and 5′ CCAGTGCATTGCCCCACTCCG), *c-Fos* (5′ TCACCCTGCCCCTTCTCA and 5′ CTGATGCTCTTGACTGGCTCC), *Cdk4* (5′ GGGGAAAATCTTTGATCTCATTGGA and 5′ AAGGCTCC TCGAGGTAGAGATA), *Vangl2* (5′ CCCCAGTTCACACTCAAGGT and 5′ ACTTGGGCAGGTTGAGGAG), and *MGF* (5′ GGAGAAGGAAAG GAAGTACATTTG and 5′ CCTGCTCCGTGGGAGGCT).

Protein determination

MuSCs were lysed on ice in 700 μL of radioimmunoprecipitation assay buffer (Sigma-Aldrich) supplemented with phosphatase (1:250; Sigma-Aldrich) and proteinase (1:50; Sigma-Aldrich) inhibitor cocktails. Cellular debris was removed by centrifugation at 13,000 rpm for 15 min at 4°C. Supernatants were transferred into fresh Eppendorf tubes and stored at −80°C. The protein concentration was determined using a Pierce BCA protein assay kit (Thermo Fisher Scientific, Waltham, MA).

Western blot

Lysates were diluted five times in Laemmli SDS buffer and denatured for 5 min at 95°C before Western blotting. Samples were electrophoresed on 4–20% Mini-PROTEAN TGX precast polyacrylamide gel (Bio-Rad) and transferred onto polyvinylidene fluoride membranes (GE Healthcare, Chicago, IL) using a wet transfer blotter (Bio-Rad). Membranes were blocked in prime blocking agent (GE Healthcare) for 1 h and incubated overnight at 4°C with primary phospho-p44/42 MAPK (Erk1/2) (Thr202/Tyr204; 1:1000; Cell Signaling Technology, Leiden, the Netherlands), p44/42 MAPK (Erk1/2) (1:1000; Cell Signaling Technology), phospho-p38 MAPK (Thr180/Tyr182; 1:3000; Cell Signaling Technology), p38 MAPK (1:2000; Cell Signaling Technology), phospho-AKT (Ser473; 1:2000; Cell Signaling Technology), AKT(pan) (1:2000; Cell Signaling Technology), α-tubulin (1:2000; Cell Signaling Technology), and pan-actin (1:1000; Cell Signaling Technology). Polyvinylidene fluoride membranes were washed three times with Tris-buffered saline containing Tween and incubated with horseradish peroxidase-conjugated anti-rabbit IgG secondary antibody (1:2000; Roche, Basel, Switzerland) before fluorescent imaging. Densities of the bands from blot images were normalized to the loading control by densitometric analysis using ImageJ software.

Statistical analysis

The paired sample *t*-test was used to test statistically significant differences in MuSC deformation data, the independent *t*-test to test differences in tensile strain data, and the one-sample *t*-test to test differences in shear strain data. Differences in NO production data were tested using analysis of variance. One-sample *t*-test was performed for statistical analysis of gene expression data, and independent *t*-test was performed to determine the difference in protein expression data. Data were expressed as mean $\pm$ SEM, and $p < 0.05$ was considered significant.

RESULTS

Ex vivo myofiber stretch induced MuSC deformation

We characterized MuSCs in their niche, within an isolated myofiber bundle from the EDL and SO muscles (Fig. 1). Sarcomere length was ~2.5 μm, slightly above the slack length of sarcomeres as described earlier (Fig. 2 *A*; (37)). After stretching of EDL myofibers up to ~40% above their slack length, MuSCs on the host myofiber increased in length by the same magnitude (Figs. 1, *A–D*, and 2 *C*). Similar increases in sarcomere length and MuSC were observed for myofiber bundles from SO muscles (Fig. 1, *I–L*). MuSCs on EDL myofibers displayed clear cell protrusions, whereas those on SO myofibers did not. Mean tensile strain on MuSCs of stretched EDL and SO myofibers was 43%, which was of the same order of magnitude as the increase in strain of the myofibers (~40%; Fig. 2 *B*). In addition to lengthening of MuSCs, filopodia were reoriented in the direction of the stretched myofibers, as indicated by the change in position of the processes when the myofibers were stretched compared with slack length (Fig. 1, *E* and *F*). MuSCs of SO myofibers showed less distinct processes compared with MuSCs of EDL myofibers (Fig. 1, *M* and *N*). A comparison of the displacement of the lateral sides of the MuSCs in the *YZ* plane and changes in the angles between the two lateral sides showed that the MuSCs were also subjected to shear deformation (Fig. 1, *G*, *H*, *O*, and *P*). The displacement of the lateral sides of MuSCs differed by ~40%, and the shear angle showed a 12° displacement from a fixed axis in both EDL and SO myofibers (Fig. 2, *D* and *E*).

Stretching of both EDL and SO myofiber bundles induced MuSC compression and decreased MuSC width by ~21% (Fig. 2 *F*). Changes in cell morphology after stretch indicated an increase in MuSC aspect ratio, as MuSCs became more elongated and a negative trend in roundness was noted (Fig. 2, *G* and *H*). These results showed that MuSCs within their native niche were subjected to tensile and shear forces causing corresponding deformations.

Fluid shear stress induced MuSC deformation

Muscle contraction can produce areas of high-pressure gradients, whereas variations in pressure gradients likely cause interstitial fluid movement. We further assessed whether MuSCs subjected to fluid flow-induced forces would undergo deformation. MuSCs were stained for F-actin and nucleus, and live-cell confocal images were acquired while MuSCs were subjected to mechanical loading by CFSS. Cross-sectional (*XZ*) time-lapse images showed that within 1 s of CFSS, the MuSC deformed in the direction of the flow (Fig. 3). The MuSC deformation was not transient, as the cell did not return to its initial state after 5 s (Fig. 3). These results showed that MuSCs deform when subjected to fluid shear forces in a parallel-plate flow chamber.

FEM of fluid shear forces on MuSC

To quantify the fluid shear forces induced by the fluid flow on MuSCs and determine the solid mechanics of a MuSC over time, FEM of PFSS in a parallel-plate fluid chamber on an adherent half-sphere was employed. The half-sphere exhibited similar cell volume, apex height, and area to the MuSCs in culture. FEM showed that the distribution of fluid pressure on the surface of the half-sphere varied substantially (Fig. 4 *A*). At 0 s, the fluid pressure was highest, i.e., 141 Pa. This value dropped to 37 Pa within 10 ms of PFSS application (Fig. 4 *B*). During PFSS treatment, the highest fluid pressure was 68 Pa at 0.25 s, after which this value dropped to 0 Pa at 0.75 s and inclined to 67 Pa at 1.2 s of PFSS application (Fig. 4 *B*).

We further predicted the fluid shear stress distribution over the MuSC during PFSS treatment. Our result showed a nonuniform distribution of fluid shear stress over the surface of the half-sphere, with the highest shear stress at the apex of this half-sphere (Fig. 4 *C*). The fluid shear stress values increased to 1.2 Pa within 0.26 s of PFSS application, followed by a drop to almost 0 at 0.75 s of PFSS treatment (Fig. 4 *D*). This was followed by an incline in shear values to 1.2 Pa at 1.2 s (Fig. 4 *D*).

To compute the areas of stress distribution over MuSCs subjected to PFSS, we performed FEM of Von Mises stress

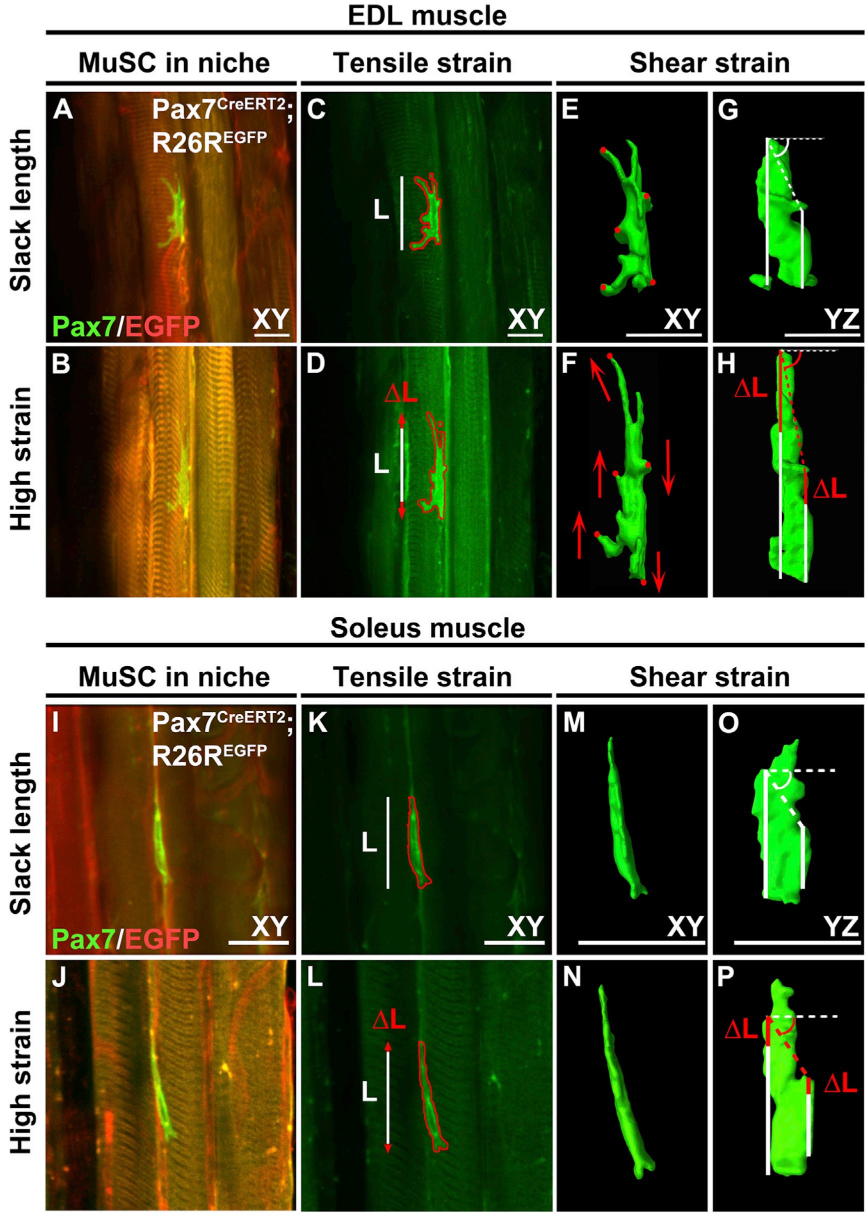

FIGURE 1 Live-cell imaging of MuSCs within their niche at slack length and high-strain myofiber bundles. (*A*, *B*, *I*, and *J*) Micrographs of typical MuSCs in their native niche within myofiber bundle at slack length and at high strain (~40% above slack length) from EDL and SO muscles. (*C*, *D*, *K*, and *L*) Measurement of MuSC length change in the *XY* plane. White lines: length of MuSCs at myofiber bundle slack length. Red arrows: increase in length at extended myofiber bundle length. (*E–H* and *M–P*) 3D reconstructions of MuSCs at slack length and high myofiber strain illustrating MuSC shear deformations in the *XY* and *YZ* planes. Stretching of the myofiber bundles caused displacement of cell regions (*red circles*) in the direction indicated by the arrows (*red*) in the *XY* plane, implicating shearing of MuSC (*E* and *F*). (*G*, *H*, *O*, and *P*) Differences in MuSC length changes at both lateral sides indicate an increase in shear angle (i.e., angle of the *dashed lines* in *YZ* plane). Scale bars, 20 μm. To see this figure in color, go online.

over the half-sphere. Our results showed nonuniform and localized areas of high stress during PFSS application (Fig. 4 *E*). The areas of highest stress induction were localized at the surface against the fluid flow and at the base of the half-sphere (Fig. 4 *E*). Von Mises stress values ranged between 5 and 38 Pa, which increased over time during fluid flow treatment and declined at the end of the fluid flow pulse at 1 s (Fig. 4 *F*).

Next, we determined PFSS-induced MuSC displacement and/or deformation, presented here as solid displacement. PFSS treatment resulted in a localized solid displacement of the half-sphere with areas of high and low displacement values, which were distributed, respectively, around the apex and in the area facing the direction of the flow (Fig. 4 *G*). The solid displacement values ranged from 0 to 9 nm between 0 and 0.8 s of PFSS application (Fig. 4 *H*).

MuSCs were mechanosensitive to PFSS

Mechanical force-induced deformation of cell shape may induce specific molecular responses by MuSCs, including elevated NO production. To measure the NO response of MuSCs treated with mechanical loading by fluid shear stress and quantify the mRNA expression levels, we adopted the PFSS in vitro model system. Within the MuSC in situ system, the NO produced by myofibers and MuSCs cannot be discriminated. Moreover, very few MuSCs are present per myofiber, resulting in very low levels of NO produced

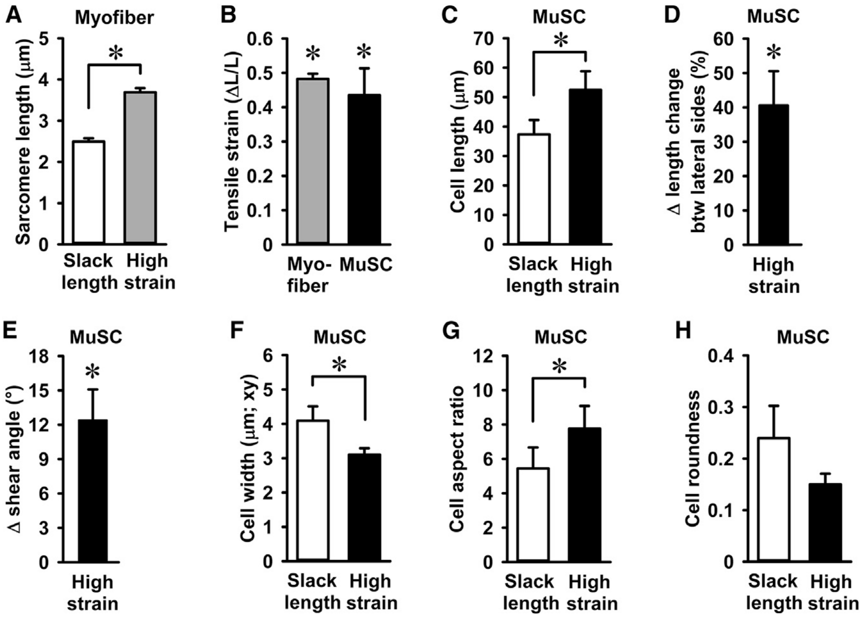

FIGURE 2 Quantification of myofiber stretch-induced compression, strain, and shearing of MuSCs. (*A*) Increase in sarcomere length upon EDL and SO myofiber bundle stretch. (*B*) Myofiber bundle sarcomere strain and MuSC strain were similar. (*C*) MuSC length at myofiber bundle slack length and high strain. (*D*) MuSCs experienced shear strain upon myofiber bundle stretch. (*E*) At high strain, the shear angle between the two lateral sides of the cell also changed by 12°. (*F–H*) Myofiber stretch decreased cell width and increased MuSC aspect ratio but did not affect MuSC roundness. n = 6–8 cells. Btw, between. *Significant effect of stretch, $p < 0.05$.

(below the detection limit of the Griess assay) by the MuSCs. Therefore, the MuSC in situ model is not suitable for measuring NO production by the MuSCs, and the PFSS in vitro model was used instead to determine the NO response by MuSCs to mechanical loading. We first determined whether PFSS caused detachment of MuSCs from the substratum. Micrographs after static and PFSS treatment indicated that ~15% of MuSCs detached after PFSS treatment, whereas no difference was observed in the static controls (Fig. 5 *A*). As NO is produced by NOS, we assessed whether MuSCs express the different isoforms of NOS (*iNOS*, *eNOS*, and *nNOS*). We found that myoblasts express *nNOS* and *iNOS*, but *eNOS* transcripts were not detectable (Fig. 5 *B*). PFSS did not affect the expression of *nNOS*, but an upward trend was seen for *iNOS* mRNA levels after PFSS treatment (Fig. 5 *B*). To assess NO production by MuSCs, NO levels in the culture medium were quantified. PFSS resulted in increased NO production by MuSCs (2- to 2.5-fold) after 10, 30, and 60 min (Fig. 5 *C*). These results indicated that MuSCs were sensitive to PFSS and that NO production was most likely related to alterations in nNOS and/or iNOS enzyme activity.

PFSS upregulated *c-Fos* and *Cdk4* with no effect on *MyoD* and *Myog*

To assess whether PFSS induces alterations in expression of genes involved in regulating MuSC proliferation and differentiation, we quantified *c-Fos*, *Cdk4*, *MyoD*, and *Myog* mRNA expression. PFSS induced a significant increase in mRNA expression of *c-Fos* by fourfold and *Cdk4* by 1.3-fold (Fig. 6 *A*). MuSCs expressed both *MyoD* and *Myog*, with no difference in expression levels between PFSS-treated and static control cells (Fig. 6 *A*). These results showed that PFSS induced expression of *c-Fos* and *Cdk4*, known to promote cell proliferation (38,39).

PFSS upregulated expression of *IL-6*, but not *COX2* and *MGF*, in myoblasts

Exercise induces *IL-6* expression in myofibers, which is involved in myoblast proliferation and regeneration (40). IL-6 production is stimulated by both NO and COX2-mediated prostaglandin production (41). COX2 and mechano

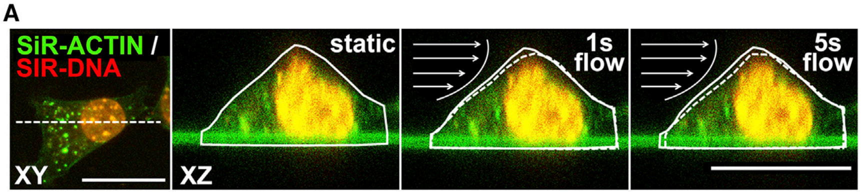

FIGURE 3 Fluid shear stress induced MuSC deformation in a parallel-plate flow chamber. Confocal top view (*XY*) and cross-sectional (*XZ*) live-cell images are given of a MuSC stained for F-actin (*green*) and nucleus (*red*), before (*static*) and during (1, 5 s) fluid shear stress treatment, illustrating the fluid shear stress-induced MuSC deformation. The change in cell morphology is illustrated by solid and dotted lines in *XZ* images. Arrows indicate the direction of fluid flow. Scale bars, 20 μm. To see this figure in color, go online.

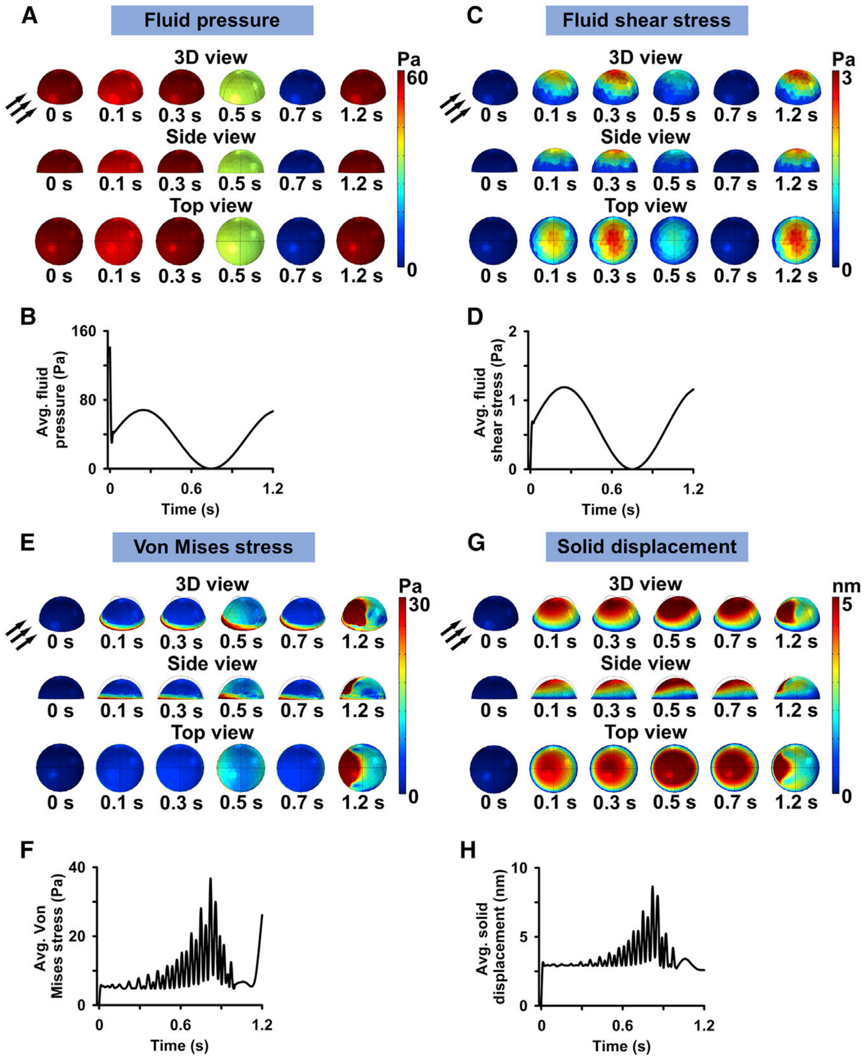

FIGURE 4 FEM of PFSS in a parallel-plate flow chamber showing the variable distribution of fluid dynamics (fluid pressure and fluid shear stress) on an adherent MuSC and solid mechanics of this cell over time. (*A*) FEM of fluid pressure on a MuSC during one pulse illustrated by 3D view, side view, and top view images. (*B*) The highest fluid pressure was detected at 0.25 s, and the lowest at 0.75 s. The average fluid pressure on the cell at each pulse was oscillating between 0 and 68 Pa and the average fluid shear stress between 0 and 1.2 Pa. (*C*) Fluid shear stress on a MuSC during one pulse illustrated by 3D view, side view, and top view images. (*D*) The highest shear stress magnitude was detected at 0.25 s and the lowest at 0.75 s. The average fluid shear stress magnitude on the cell at each pulse was oscillating between 0 and 1.2 Pa. (*E*) FEM of Von Mises stress of a MuSC during one pulse illustrated by 3D view, side view, and top view images. (*F*) Von Mises stress ranged from 5 to 38 Pa. (*G*) FEM of solid displacement of a MuSC during one pulse illustrated by 3D view, side view, and top view images. (*H*) Solid displacement ranged from 0 to 9 nm. Black arrows, fluid flow direction; surface color, magnitude. To see this figure in color, go online.

growth factor (MGF) are also essential for myoblast proliferation and differentiation (42,43). Therefore, we determined the effect of PFSS on *IL-6*, *COX2*, and *MGF* expression in MuSCs. PFSS induced a significant twofold increase in *IL-6* expression and an upward trend toward increased *COX2* expression but did not affect *MGF* expression (Fig. 6 *B*).

PFSS did not affect Wnt signaling

Wnt1 has been shown to stimulate MuSC proliferation (44). To investigate the effect of PFSS on Wnt signaling within MuSCs, we evaluated the effect of PFSS on components of the Wnt signaling pathway (Wnt1, Wnt3a, and Wnt5a) and regulators of Wnt signaling, i.e., Rspo1 and Dickkopf-related protein 1 (Dkk1) (45). PFSS did not affect *Wnt1* gene expression, whereas an upward trend was seen in *Wnt5a* mRNA levels (Fig. 6 *C*). *Wnt3a* was undetectable, and *Rspo1*, a positive regulator of Wnt activity, was unchanged by PFSS, whereas *Dkk1*, a negative regulator, was undetectable (Fig. 6 *C*). These results indicated that the Wnt signaling pathway was not affected by mechanical stimuli in MuSCs.

PFSS did not alter expression of PCP genes

Wnt5a is implicated in mediating membrane protein Vangl2 phosphorylation and activation (46), known to affect the PCP pathway that is crucial for cell division and cell migration. We determined whether PFSS induced expression of *Vangl2* and showed that *Vangl2* expression was not affected by PFSS treatment (Fig. 6 *D*). We further tested the effect of PFSS on *Wnt7a* and *Wnt10b* expression, which are involved in self-renewal and myogenic commitment of MuSCs. *Wnt7a* transcripts were not detectable in MuSCs, and *Wnt10b* expression was not affected by PFSS (Fig. 6 *D*). Therefore, PFSS did not affect expression of genes involved in PCP formation in MuSCs.

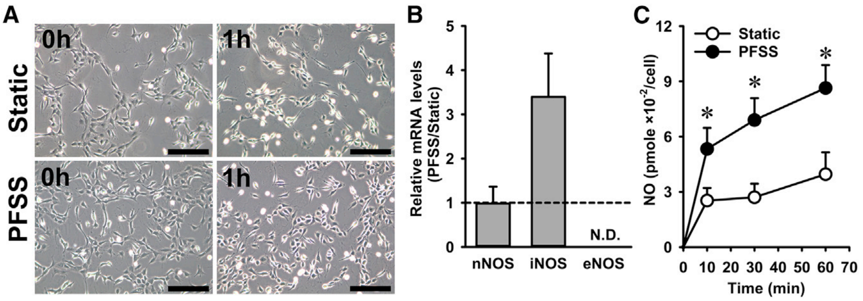

FIGURE 5 PFSS induced NO production and elevated *iNOS* expression in primary MuSCs. (*A*) Micrographs of cultured primary mouse MuSCs at 1 h post-static and PFSS treatment indicate ~15% loss of cells because of PFSS application. (*B*) PFSS did not change the expression of *nNOS*, whereas *eNOS* was not detectable, and PFSS caused an upward trend in the level of *iNOS* mRNA transcripts. (*C*) PFSS induced NO production at 10, 30, and 60 min by 2- to 2.5-fold. n = 20. ND, not detectable. *Significant effect of PFSS, $p < 0.05$. Scale bars, 200 μm.

PFSS-induced activation of ERK and p38 MAPK

MuSCs responded to PFSS by increased NO production and changes in gene expression (see earlier). We further asked whether PFSS also influences MAPK signaling and determined the effect of PFSS on ERK MAPK activation. Our results showed a significant fivefold increase in phosphorylation of ERK during 1 h PFSS treatment compared with static controls, but no difference in total ERK protein levels was observed (Fig. 7 *A*). PFSS also induced a threefold increase in p38 phosphorylation with no effect on total p38 levels (Fig. 7 *B*). We further investigated changes in AKT signaling by determining AKT phosphorylation in static control and PFSS-treated MuSCs. PFSS induced a slight, but not significant, increase in AKT phosphorylation (1.6-fold), whereas no difference in total AKT levels was observed (Fig. 7 *C*).

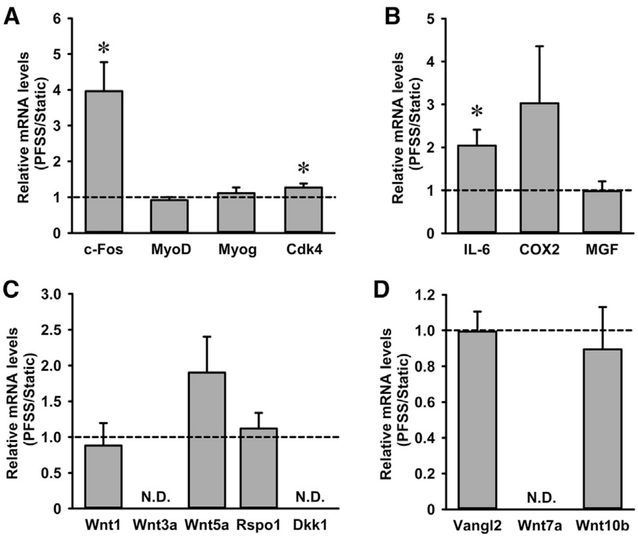

FIGURE 6 PFSS upregulated proliferation marker expression, i.e., *c-Fos*, *Cdk4*, and *IL-6*, in primary mouse MuSCs. (*A*) PFSS upregulated *c-Fos* expression by fourfold and *Cdk4* expression by 1.3-fold but did not affect *MyoD* and *Myog* mRNA expression. (*B*) *IL-6* was upregulated by twofold after PFSS, and an upward trend was observed in *COX2* expression, whereas no affect was shown on *MGF* by PFSS. (*C*) *Wnt5a* transcripts were slightly, but not significantly, upregulated, *Wnt1* and *Rspo1* expression remained unchanged, whereas *Wnt3a* and *Dkk1* were undetectable in both static and PFSS-treated cells. (*D*) PFSS did not affect *Vangl2* and *Wnt10b* expression, whereas *Wnt7a* was not detectable. n = 7–10. ND, not detectable. *Significant effect of PFSS, $p < 0.05$.

DISCUSSION

MuSCs within their native niche on the host myofiber with an intact endomysium are presumed to undergo deformations when the myofibers are subjected to tensile strain. Here, we have shown for the first time, to our knowledge, that MuSCs underwent both tensile strain and shear loading in response to myofiber strain. Because of the constancy of myofiber volume, the endomysium surrounding the myofiber will undergo a reorientation from a longitudinal toward a more radial orientation upon muscle contraction (47). Because MuSCs are anchored to the basal lamina and the sarcolemma of the host myofiber, one may expect that these cells also align in the orientation of basal lamina. Our data confirm such reorientation of MuSCs with respect to the longitudinal axis of the myofibers.

Local differences in the stiffness of the endomysium may occur because of the presence of capillaries and differences in type of collagen and/or connective tissue content. These differences will cause shear loads and shear deformation of MuSCs upon mechanical loading of skeletal muscle. In both myofiber bundles of EDL and SO, we observed differences in lateral displacements along the length of MuSCs. This indicates that MuSCs are subjected to shear loading during myofibers stretch-shortening cycles. The MuSCs on the SO myofibers showed slightly less protrusions compared with the MuSCs on the EDL myofibers, likely as a result of differences in ECM composition and/or expression of sarcolemma proteins between the two myofiber types. SO myofibers of rat and mouse muscles express higher levels of integrin β1 compared with EDL myofibers (48). Moreover, transcriptome analysis shows different expression levels of various skeletal muscle-specific genes between EDL and SO muscles (49,50). However, more research is needed to draw a conclusive inference about the difference between ECM composition of EDL and SO muscles, as well as in sarcolemma transmembrane protein expression in these muscles. Mechanical loading of skeletal muscle may also cause localized changes in intramuscular pressure because of the movement of interstitial fluid, thus creating regions

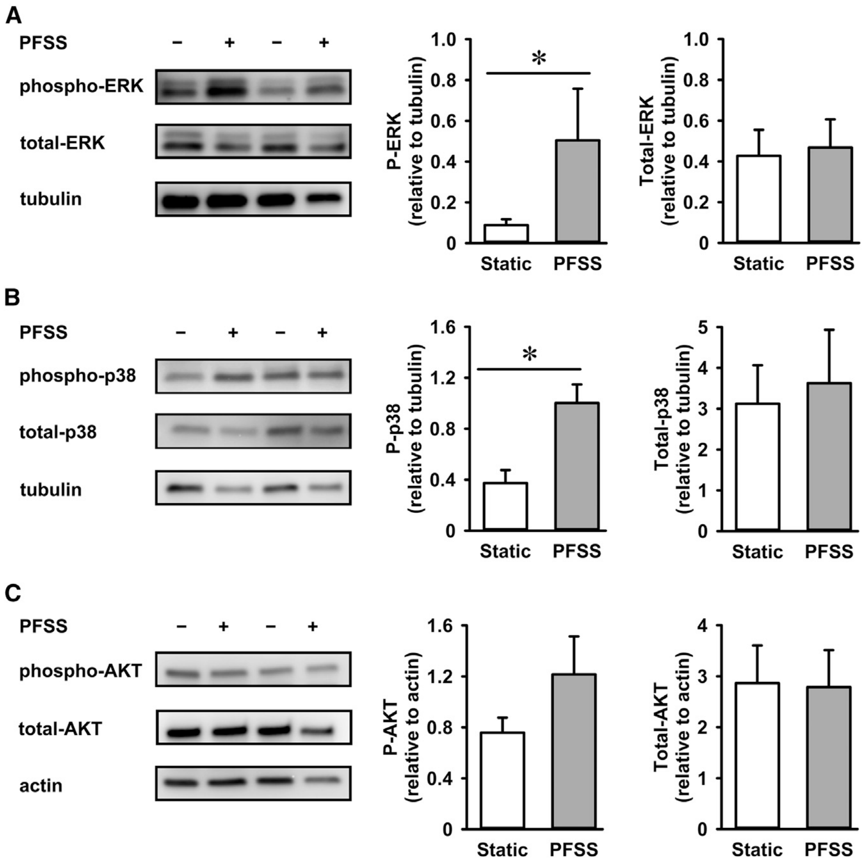

FIGURE 7 PFSS treatment induced MAPKs ERK 1/2 and p38 phosphorylation and activation. (*A*) Expression of ERK 1/2 in MuSCs and PFSS-induced changes in the phosphorylation levels of ERK 1/2. Values are normalized to α-tubulin expression levels. (*B*) Expression of p38 in MuSCs and PFSS-induced changes in the phosphorylation levels of p38. Values are normalized to α-tubulin expression levels. (*C*) Expression levels of AKT and phospho-AKT in static and PFSS-treated MuSCs. Values are normalized to actin expression levels. *Significant effect of PFSS, $p < 0.05$.

of different pressure gradients (11). Further studies are required to determine whether these pressure gradients potentially cause MuSC compression and result in altered cellular osmotic pressure and volume of MuSCs due to water efflux (51). Osmotic pressure is a well-known regulator of mechanotransduction, but whether MuSC deformation by myofiber stretching induces activity in this manner still needs to be determined. This study provides the proof of concept that MuSCs are subjected to forces and undergo different mechanical deformations, which encourages further research on the nature of these forces in MuSC function and muscle regeneration.

Mechanical forces are inherent to skeletal muscles, as the myofibers are subjected to stretch and shear forces. Our results showed that myofiber stretch induced tensile and shear forces within myofibers that can propagate to the MuSCs. Stretching of MuSCs results in the production of factors involved in MuSC activation, self-renewal, and proliferation (52). Furthermore, pulsatile shear forces are known to elicit mechanotransduction in various cell types, e.g., endothelial cells, mesenchymal stem cells, and bone cells (53). We investigated whether shear forces can elicit mechanotransduction events within MuSCs and alter gene expression and MAPK signaling. We adopted the 1 Hz pulsed frequency based on the stride frequency of human gait, which ranges between 0.8 and 1.26 Hz during normal walking (34). Live-cell imaging of MuSCs subjected to CFSS showed that MuSCs underwent deformation. However, the extent of deformation was less compared with that of MuSCs within their niche. To quantify the fluid flow-induced forces on MuSCs in the parallel-plate flow chamber, we performed FEM. MuSCs subjected to PFSS were acted upon by various mechanical loads such as fluid pressure, fluid shear stress, and Von Mises stress. FEM illustrated that MuSCs underwent fluid flow-induced deformation, presented as solid displacement.

Our results showed that a small subset of MuSCs were detached after PFSS treatment. These MuSCs were likely loosely attached to the matrigel-coated substrate or were undergoing apoptosis. No difference was seen in static MuSCs. We observed that shear loading of MuSCs induced NO production and upregulated expression of *c-Fos*, *Cdk4*, and *IL-6*. NO is known to release hepatocyte growth factor from the ECM and to mediate MuSC activation, proliferation, and fusion (52). Because we showed that both *nNOS* and *iNOS* mRNA are expressed in MuSCs under static conditions, it is likely that PFSS-induced NO upregulation results from changes in the nNOS and/or iNOS enzyme activity. *c-Fos* is an early response gene and plays an important role in cell growth, proliferation, and differentiation (54). During

differentiation, c-Fos is repressed by MyoD and Myog (55,56). c-Fos is a subunit of transcription factor complex activator protein-1 (AP-1) (57), which binds to the *MyoD* promotor and inhibits *MyoD* transcription in proliferating myoblasts (58). Overexpression of c-Fos has been shown to promote proliferation and inhibit differentiation in C2C12 myoblasts (59). This suggests that increased *c-Fos* expression after shear loading may induce MuSC proliferation. Moreover, CDK4 has been shown to play a role in cell cycle progression together with D-type cyclins (39). IL-6 is essential for muscle regeneration and MuSC proliferation (40). Muscle injury causes IL-6 production by macrophages and myofibers (4). In this study, increased *IL-6* expression by shear-loaded MuSCs suggests a positive effect of loading on MuSC proliferation and indicates that IL-6 may act in both a paracrine as well as an autocrine manner to induce MuSC proliferation. Moreover, expression of *COX2*, which is induced in response to injury and is involved in prostaglandin production and MuSC proliferation, also showed an upward trend after shear loading. Another important factor driving MuSC differentiation is MGF, which is induced in myotubes by PFSS (29). We did not observe changes in *MGF* expression in MuSCs because of PFSS, suggesting that mechanical loading for 1 h does not induce autocrine MGF signaling, whereas it does in differentiated myotubes (29). This suggests that in addition to muscle damage and mechanical stretch, which stimulate MuSC proliferation, here we show that PFSS induced expression of proliferation-related genes and likely stimulates MuSC proliferation.

Shear loading did not affect the expression of genes associated with differentiation of myoblasts, as indicated by unaltered *MyoD* and *Myog* expression. Members of the Wnt family also govern MuSC function. Exogenous Wnt1 activates *Myf5* expression, resulting in MuSC activation and/or proliferation, as shown in rhabdomyosarcomas (60,61). Moreover, Wnt3a is required for both myogenic potential and differentiation of MuSCs (62). We show that that *Wnt1* mRNA levels were unaltered in response to PFSS in MuSCs, whereas *Wnt3a* was not detectable in both controls and PFSS-treated cells. Wnt5a overexpression has also been shown to increase MuSC proliferation (44). We show that MuSCs upregulate *Wnt5a* mRNA levels in response to PFSS, but the increase did not reach statistical significance. Wnt signaling may also be regulated by negative or positive mediators. Rspo1 mediates canonical Wnt signaling, thereby affecting MuSC differentiation (63). However, we did not observe changed *Rspo1* expression by PFSS. *Dkk1*, a negative regulator of Wnt signaling, was not detectable in MuSCs, suggesting that there was only a limited response by canonical Wnt signaling to PFSS, possibly involving upregulation of *Wnt5a*.

Wnt7a induces symmetric expansion of MuSCs by activating the PCP pathway through regulation of Vangl2 and is negatively regulated by Rspo1 (45,64). Wnt10b is involved in determining myogenic commitment of MuSCs, as its ablation has been shown to decrease MuSC myogenicity (65). We did not detect *Wnt7a* expression in MuSCs, and *Vangl2* and *Wnt10b* expression was unaffected by shear loading. These results suggest that PFSS did not affect mRNA expression of genes involved in the PCP pathway, and further research is needed using immunostaining to determine possible changes in the spatial distribution of PCP components in MuSCs in their niche attached to an intact basal lamina. Cultured MuSCs are attached only to the matrigel-coated culture dish, whereas MuSCs within their natural niche are linked to the sarcolemma and basal lamina. These linkages are essential for the determination of PCP protein and receptor distribution. Moreover, the effect of mechanical loading on Fzd receptor expression, as well as canonical and noncanonical Wnt signaling, is interesting but needs further investigation.

We were able to show that 1 h PFSS resulted in phosphorylation and activation of ERK 1/2 and p38 MAPKs. In bone cells, integrins play an important role in fluid shear stress-induced ERK and p38 MAPK signaling and in the expression of bone-formation-related genes (17). MuSCs in their niche are anchored to the basal lamina and sarcolemma via transmembrane receptors, and MuSC shearing likely initiates downstream signaling and MAPK activation via these receptors. However, within the PFSS flow chamber, MuSCs were attached to a matrigel substrate, and the fluid shear stress-induced activation of ERK 1/2 and p38 likely occurred via other mechanisms. Our results suggested that fluid shear stress was a potent activator of MAPK signaling. During myofiber stretch, the shearing of the MuSCs in their niche, as well as interstitial fluid movement, likely induces downstream signaling pathways, which are involved in MuSC activation, proliferation, and diverse other cellular functions. Our results are in line with results from a previous study in which MuSCs were subjected to cyclic stretch resulting in ERK activation (66). However, unlike this previous study (66), we showed that PFSS induced p38 activation. MuSCs subjected to cyclic stretch in the presence of an ERK inhibitor show declined proliferation and increased expression of differentiation factors (66). Moreover, fluid shear stress-induced AKT activation in bone cells depends on NO production (67). We showed that PFSS did induce NO production, but it did not activate AKT within 1 h of PFSS.

CONCLUSIONS

We conclude that MuSCs experience tensile strain, shear strain, and pressure in response to mechanical perturbations of the myofibers to which they are attached and that mechanotransduction is likely to occur in vivo in MuSCs during muscle stretch-shortening. Shear loading of MuSCs in vitro upregulated a number of genes as well as activated ERK 1/2 and p38 MAPK signaling, which are known to promote MuSC proliferation (Fig. 8, *A* and *B*). Fate mapping of

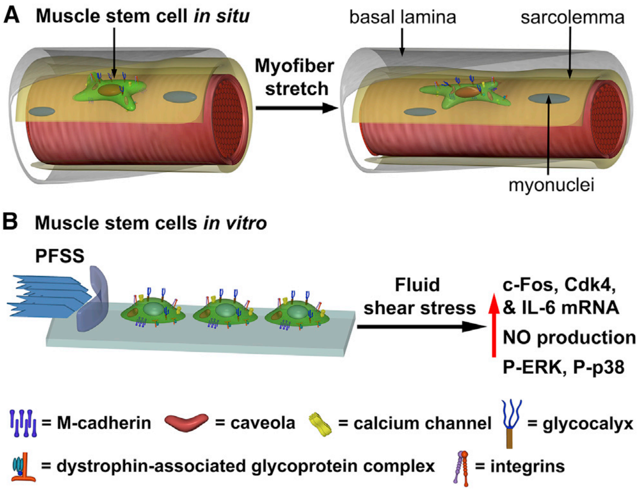

FIGURE 8 Schematic of myofiber stretch inducing MuSC deformation, and effect of fluid shear stress on myoblast mRNA expression. (*A*) MuSC in its niche on the host myofiber, embedded between the sarcolemma (*yellow*) and basal lamina (*gray*) and connected via transmembrane complexes, undergoing tensile strain and shear stress deformations upon myofiber stretch. (*B*) Myoblasts subjected to mechanical load by PFSS in vitro induce upregulation of NO and expression of factors known to promote proliferation. To see this figure in color, go online.

MuSCs subjected to PFSS will further reveal whether this acts as a cue to induce MuSC proliferation and/or differentiation. Note that we observed that stretching of MuSCs also affected their cellular processes, thus potentially affecting their ability to migrate. Extended time-lapsed imaging of cells will therefore reveal how exposure to tensile and shear strain regulates MuSC proliferation, differentiation, and migration. This will provide important insight into how MuSCs respond to such forces in the context of aging and disease and how this affects their ability to maintain effective muscle function.

SUPPORTING MATERIAL

Supporting material can be found online at https://doi.org/10.1016/j.bpj.2021.05.021.

AUTHOR CONTRIBUTIONS

M.H., J.J., K.J.L., R.D.K., and R.T.J. performed the experiments. M.H., A.D.B., C.O., H.S., G.M.J.d.W., and R.D.K. analyzed the data. M.H., J.J., and R.T.J. drafted the manuscript. M.H., J.K.-N., A.D.B., J.J., C.O., F.L.G., L.G., K.J.L., R.D.K., H.S., G.M.J.d.W., and R.T.J. contributed to conceiving and planning the experiments, interpreted the results, and edited and revised the manuscript.

ACKNOWLEDGMENTS

The authors thank Dylan Herzog for technical assistance.

R.D.K. and R.T.J. were funded by a Royal Society International Partnership Award (IE150196). K.J.L. was funded by the BBSRC (BB/R015953/01) and the British Heart Foundation. R.D.K. was funded by the Wellcome Trust (101529/Z/13/Z). F.L.G. was supported by grants from Agence Nationale pour la Recherche (ANR-14-CE11-0026) and Association Française contre les Myopathies/AFM Telethon. J.J. was funded by the China Scholarship Council (no. 201608530156). M.H. was funded by the European Commission through MOVE-AGE, an Erasmus Mundus Joint Doctorate program (grant number: 2014-0691).

REFERENCES

1. Chakravarthy, M. V., B. S. Davis, and F. W. Booth. 2000. IGF-I restores satellite cell proliferative potential in immobilized old skeletal muscle. *J Appl Physiol (1985).* 89:1365–1379.
2. Chen, H. X., S. P. Tang, …, W. Shi. 2014. Fibrosis, adipogenesis, and muscle atrophy in congenital muscular torticollis. *Medicine (Baltimore).* 93:e138.
3. Van der Meer, S. F., R. T. Jaspers, and H. Degens. 2011. Is the myonuclear domain size fixed? *J. Musculoskelet. Neuronal Interact.* 11:286–297.
4. Bentzinger, C. F., Y. X. Wang, …, M. A. Rudnicki. 2013. Cellular dynamics in the muscle satellite cell niche. *EMBO Rep.* 14:1062–1072.
5. Huijing, P. A., and R. T. Jaspers. 2005. Adaptation of muscle size and myofascial force transmission: a review and some new experimental results. *Scand. J. Med. Sci. Sports.* 15:349–380.
6. Peake, J. M., P. Della Gatta, …, D. C. Nieman. 2015. Cytokine expression and secretion by skeletal muscle cells: regulatory mechanisms and exercise effects. *Exerc. Immunol. Rev.* 21:8–25.
7. Kivelä, R., M. Silvennoinen, …, H. Kainulainen. 2008. Exercise-induced expression of angiogenic growth factors in skeletal muscle and in capillaries of healthy and diabetic mice. *Cardiovasc. Diabetol.* 7:13.
8. Yucesoy, C. A., B. H. Koopman, …, H. J. Grootenboer. 2002. Three-dimensional finite element modeling of skeletal muscle using a two-domain approach: linked fiber-matrix mesh model. *J. Biomech.* 35:1253–1262.
9. Jaspers, R. T., R. Brunner, …, P. A. Huijing. 1999. Acute effects of intramuscular aponeurotomy on rat gastrocnemius medialis: force transmission, muscle force and sarcomere length. *J. Biomech.* 32:71–79.
10. Jaspers, R. T., R. Brunner, …, P. A. Huijing. 2002. Acute effects of intramuscular aponeurotomy and tenotomy on multitendoned rat EDL: indications for local adaptation of intramuscular connective tissue. *Anat. Rec.* 266:123–135.
11. Evertz, L. Q., S. M. Greising, …, K. R. Kaufman. 2016. Analysis of fluid movement in skeletal muscle using fluorescent microspheres. *Muscle Nerve.* 54:444–450.
12. Yin, H., F. Price, and M. A. Rudnicki. 2013. Satellite cells and the muscle stem cell niche. *Physiol. Rev.* 93:23–67.
13. Boers, H. E., M. Haroon, …, R. T. Jaspers. 2018. —Mechanosensitivity of aged muscle stem cells. *J. Orthop. Res.* 36:632–641.
14. Wozniak, A. C., O. Pilipowicz, …, J. E. Anderson. 2003. C-Met expression and mechanical activation of satellite cells on cultured muscle fibers. *J. Histochem. Cytochem.* 51:1437–1445.
15. Tatsumi, R., S. M. Sheehan, …, R. E. Allen. 2001. Mechanical stretch induces activation of skeletal muscle satellite cells in vitro. *Exp. Cell Res.* 267:107–114.
16. Weinbaum, S., S. C. Cowin, and Y. Zeng. 1994. A model for the excitation of osteocytes by mechanical loading-induced bone fluid shear stresses. *J. Biomech.* 27:339–360.
17. Lee, D. Y., C. R. Yeh, …, J. J. Chiu. 2008. Integrin-mediated expression of bone formation-related genes in osteoblast-like cells in response to fluid shear stress: roles of extracellular matrix, Shc, and mitogen-activated protein kinase. *J. Bone Miner. Res.* 23:1140–1149.
18. Yeh, C. R., J. J. Chiu, …, C. K. Cheng. 2010. Estrogen augments shear stress-induced signaling and gene expression in osteoblast-like cells via

estrogen receptor-mediated expression of beta1-integrin. *J. Bone Miner. Res.* 25:627–639.

19. Roskoski, R., Jr. 2012. ERK1/2 MAP kinases: structure, function, and regulation. *Pharmacol. Res.* 66:105–143.
20. Lavoie, J. N., G. L'Allemain, ..., J. Pouysségur. 1996. Cyclin D1 expression is regulated positively by the p42/p44MAPK and negatively by the p38/HOGMAPK pathway. *J. Biol. Chem.* 271:20608–20616.
21. Alvarez, E., I. C. Northwood, ..., R. J. Davis. 1991. Pro-Leu-Ser/Thr-Pro is a consensus primary sequence for substrate protein phosphorylation. Characterization of the phosphorylation of c-myc and c-jun proteins by an epidermal growth factor receptor threonine 669 protein kinase. *J. Biol. Chem.* 266:15277–15285.
22. Lee, J. C., J. T. Laydon, ..., P. R. Young. 1994. A protein kinase involved in the regulation of inflammatory cytokine biosynthesis. *Nature.* 372:739–746.
23. Freshney, N. W., L. Rawlinson, ..., J. Saklatvala. 1994. Interleukin-1 activates a novel protein kinase cascade that results in the phosphorylation of Hsp27. *Cell.* 78:1039–1049.
24. Raingeaud, J., S. Gupta, ..., R. J. Davis. 1995. Pro-inflammatory cytokines and environmental stress cause p38 mitogen-activated protein kinase activation by dual phosphorylation on tyrosine and threonine. *J. Biol. Chem.* 270:7420–7426.
25. Lee, D. Y., Y.-S. J. Li, ..., S. Chien. 2010. Oscillatory flow-induced proliferation of osteoblast-like cells is mediated by alphavbeta3 and beta1 integrins through synergistic interactions of focal adhesion kinase and Shc with phosphatidylinositol 3-kinase and the Akt/mTOR/p70S6K pathway. *J. Biol. Chem.* 285:30–42.
26. Cantley, L. C. 2002. The phosphoinositide 3-kinase pathway. *Science.* 296:1655–1657.
27. Manning, B. D., and L. C. Cantley. 2007. AKT/PKB signaling: navigating downstream. *Cell.* 129:1261–1274.
28. Tidball, J. G., E. Lavergne, ..., M. Wehling. 1998. Mechanical loading regulates NOS expression and activity in developing and adult skeletal muscle. *Am. J. Physiol.* 275:C260–C266.
29. Juffer, P., A. D. Bakker, ..., R. T. Jaspers. 2014. Mechanical loading by fluid shear stress of myotube glycocalyx stimulates growth factor expression and nitric oxide production. *Cell Biochem. Biophys.* 69:411–419.
30. Buono, R., C. Vantaggiato, ..., E. Clementi. 2012. Nitric oxide sustains long-term skeletal muscle regeneration by regulating fate of satellite cells via signaling pathways requiring Vangl2 and cyclic GMP. *Stem Cells.* 30:197–209.
31. von Maltzahn, J., N. C. Chang, ..., M. A. Rudnicki. 2012. Wnt signaling in myogenesis. *Trends Cell Biol.* 22:602–609.
32. Holguin, N., M. D. Brodt, and M. J. Silva. 2016. Activation of Wnt signaling by mechanical loading is impaired in the bone of old mice. *J. Bone Miner. Res.* 31:2215–2226.
33. Brooks, S. V., and J. A. Faulkner. 2001. Severity of contraction-induced injury is affected by velocity only during stretches of large strain. *J Appl Physiol (1985).* 91:661–666.
34. Danion, F., E. Varraine, ..., J. Pailhous. 2003. Stride variability in human gait: the effect of stride frequency and stride length. *Gait Posture.* 18:69–77.
35. Bacabac, R. G., T. H. Smit, ..., J. Klein-Nulend. 2005. Dynamic shear stress in parallel-plate flow chambers. *J. Biomech.* 38:159–167.
36. Seddiqi, H., A. Saatchi, ..., J. Klein-Nulend. 2020. Inlet flow rate of perfusion bioreactors affects fluid flow dynamics, but not oxygen concentration in 3D-printed scaffolds for bone tissue engineering: computational analysis and experimental validation. *Comput. Biol. Med.* 124:103826.
37. Edman, K. A. P. 2005. Contractile properties of mouse single muscle fibers, a comparison with amphibian muscle fibers. *J. Exp. Biol.* 208:1905–1913.
38. Pai, S. R., and R. C. Bird. 1994. c-fos expression is required during all phases of the cell cycle during exponential cell proliferation. *Anticancer Res.* 14:985–994.
39. Tsutsui, T., B. Hesabi, ..., H. Kiyokawa. 1999. Targeted disruption of CDK4 delays cell cycle entry with enhanced p27(Kip1) activity. *Mol. Cell. Biol.* 19:7011–7019.
40. Serrano, A. L., B. Baeza-Raja, ..., P. Muñoz-Cánoves. 2008. Interleukin-6 is an essential regulator of satellite cell-mediated skeletal muscle hypertrophy. *Cell Metab.* 7:33–44.
41. Hinson, R. M., J. A. Williams, and E. Shacter. 1996. Elevated interleukin 6 is induced by prostaglandin E2 in a murine model of inflammation: possible role of cyclooxygenase-2. *Proc. Natl. Acad. Sci. USA.* 93:4885–4890.
42. Makris, A. C., Y. Sotzios, ..., T. Vassilakopoulos. 2010. Nitric oxide stimulates interleukin-6 production in skeletal myotubes. *J. Interferon Cytokine Res.* 30:321–327.
43. Otis, J. S., T. J. Burkholder, and G. K. Pavlath. 2005. Stretch-induced myoblast proliferation is dependent on the COX2 pathway. *Exp. Cell Res.* 310:417–425.
44. Otto, A., C. Schmidt, ..., K. Patel. 2008. Canonical Wnt signalling induces satellite-cell proliferation during adult skeletal muscle regeneration. *J. Cell Sci.* 121:2939–2950.
45. Binnerts, M. E., K. A. Kim, ..., A. Abo. 2007. R-Spondin1 regulates Wnt signaling by inhibiting internalization of LRP6. *Proc. Natl. Acad. Sci. USA.* 104:14700–14705.
46. Yang, Y. 2012. Wnt signaling in development and disease. *Cell Biosci.* 2:14.
47. Purslow, P. P., and J. A. Trotter. 1994. The morphology and mechanical properties of endomysium in series-fibred muscles: variations with muscle length. *J. Muscle Res. Cell Motil.* 15:299–308.
48. Gumerson, J. D., Z. T. Kabaeva, ..., D. E. Michele. 2010. Soleus muscle in glycosylation-deficient muscular dystrophy is protected from contraction-induced injury. *Am. J. Physiol. Cell Physiol.* 299:C1430–C1440.
49. Terry, E. E., X. Zhang, ..., M. E. Hughes. 2018. Transcriptional profiling reveals extraordinary diversity among skeletal muscle tissues. *eLife.* 7:e34613.
50. Zhu, J., X. Shi, ..., G. Yang. 2016. RNA-seq transcriptome analysis of extensor digitorum longus and soleus muscles in large white pigs. *Mol. Genet. Genomics.* 291:687–701.
51. Delarue, M., F. Montel, ..., G. Cappello. 2014. Compressive stress inhibits proliferation in tumor spheroids through a volume limitation. *Biophys. J.* 107:1821–1828.
52. Tatsumi, R. 2010. Mechano-biology of skeletal muscle hypertrophy and regeneration: possible mechanism of stretch-induced activation of resident myogenic stem cells. *Anim. Sci. J.* 81:11–20.
53. Martino, F., A. R. Perestrelo, ..., G. Forte. 2018. Cellular mechanotransduction: from tension to function. *Front. Physiol.* 9:824.
54. Milde-Langosch, K. 2005. The Fos family of transcription factors and their role in tumourigenesis. *Eur. J. Cancer.* 41:2449–2461.
55. Trouche, D., M. Grigoriev, ..., A. Harel-Bellan. 1993. Repression of c-fos promoter by MyoD on muscle cell differentiation. *Nature.* 363:79–82.
56. Trouche, D., H. Masutani, ..., A. Harel-Bellan. 1995. Myogenin binds to and represses c-fos promoter. *FEBS Lett.* 361:140–144.
57. Curran, T., and B. R. Franza, Jr. 1988. Fos and Jun: the AP-1 connection. *Cell.* 55:395–397.
58. Pedraza-Alva, G., J. M. Zingg, and J. P. Jost. 1994. AP-1 binds to a putative cAMP response element of the MyoD1 promoter and negatively modulates MyoD1 expression in dividing myoblasts. *J. Biol. Chem.* 269:6978–6985.
59. Hou, L., L. Zhu, ..., C. Wang. 2019. MiR-501–3p forms a feedback loop with FOS, MDFI, and MyoD to regulate c2c12 myogenesis. *Cells.* 8:573.
60. Williamson, D., J. Selfe, ..., J. Shipley. 2007. Role for amplification and expression of glypican-5 in rhabdomyosarcoma. *Cancer Res.* 67:57–65.

61. Tajbakhsh, S., U. Borello, …, G. Cossu. 1998. Differential activation of Myf5 and MyoD by different Wnts in explants of mouse paraxial mesoderm and the later activation of myogenesis in the absence of Myf5. *Development.* 125:4155–4162.

62. Tanaka, S., K. Terada, and T. Nohno. 2011. Canonical Wnt signaling is involved in switching from cell proliferation to myogenic differentiation of mouse myoblast cells. *J. Mol. Signal.* 6:12.

63. Lacour, F., E. Vezin, …, F. Le Grand. 2017. R-spondin1 controls muscle cell fusion through dual regulation of antagonistic Wnt signaling pathways. *Cell Rep.* 18:2320–2330.

64. Le Grand, F., A. E. Jones, …, M. A. Rudnicki. 2009. Wnt7a activates the planar cell polarity pathway to drive the symmetric expansion of satellite stem cells. *Cell Stem Cell.* 4:535–547.

65. Vertino, A. M., J. M. Taylor-Jones, …, C. A. Peterson. 2005. Wnt10b deficiency promotes coexpression of myogenic and adipogenic programs in myoblasts. *Mol. Biol. Cell.* 16:2039–2048.

66. Kook, S. H., Y. O. Son, …, J. C. Lee. 2008. Cyclic mechanical stress suppresses myogenic differentiation of adult bovine satellite cells through activation of extracellular signal-regulated kinase. *Mol. Cell. Biochem.* 309:133–141.

67. Rangaswami, H., R. Schwappacher, …, R. B. Pilz. 2012. Protein kinase G and focal adhesion kinase converge on Src/Akt/β-catenin signaling module in osteoblast mechanotransduction. *J. Biol. Chem.* 287:21509–21519.

Biophysical Journal, Volume *120*

Supplemental information

Myofiber stretch induces tensile and shear deformation of muscle stem cells in their native niche

Mohammad Haroon, Jenneke Klein-Nulend, Astrid D. Bakker, Jianfeng Jin, Hadi Seddiqi, Carla Offringa, Gerard M.J. de Wit, Fabien Le Grand, Lorenzo Giordani, Karen J. Liu, Robert D. Knight, and Richard T. Jaspers

Supplemental Information

MS title: Myofiber stretch induces tensile and shear deformation of muscle stem cells in their native niche

Authors: M Haroon, J Klein-Nulend, AD Bakker, J Jin, H Seddiqi, C Offringa, GMJ de Wit, F Le Grand, L Giordani, KJ Liu, RD Knight, RT Jaspers

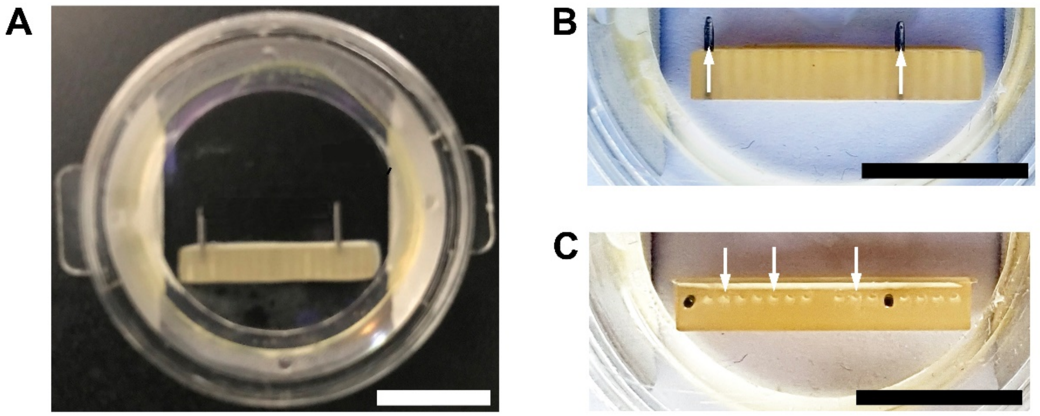

Figure S1. Chamber for incubation of myofiber bundle at slack length or high strain, consisting of a glass bottom and adjustable platinum rods. (A) Chamber used to maintain the myofiber bundle at slack length or high strain. (B) The platinum rods (arrows) in the chamber used to mount the myofiber bundles. (C) The inserts (arrows) in the bar holder allow to maintain the myofiber bundle at slack length or high strain by adjusting the distance between the rods. Scale bar, 1 cm.
